# Partial Atrioventricular Septal Defect with Left Atrioventricular Valve Aneurysm Mimicking Valve Perforation

**DOI:** 10.21470/1678-9741-2022-0218

**Published:** 2023-06-14

**Authors:** Marien Lenoir, Holy Ranaivoson, Anne Claire Casalta, Loïc Macé, Philippe Aldebert, Alexis Theron

**Affiliations:** 1 Department of Cardiac Surgery, Hôpital de la Timone, Assistance Publique des Hôpitaux de Marseille, Marseille, France; 2 Department of Cardiology, Centre Hospitalier Universitaire Tambohobe Fianarantsoa, Antananarivo, Madagascar; 3 Department of Cardiology, Hôpital de la Timone, Assistance Publique-Hôpitaux de Marseille, Marseille, France

**Keywords:** Partial Atrioventricular Septal, Mitral Valve, Aneurysm, Mitral Valve, Mitral Valve Cleft

## Abstract

Left atrioventricular valve aneurysm is a rare condition. Here we present a rare case of partial atrioventricular septal defect with an extremely thin left atrioventricular valve aneurysm mimicking valve perforation. Preoperative echocardiography demonstrated severe left sided atrioventricular valve regurgitation on the “cleft” and leaflet perforation. But we discovered a left sided atrioventricular valve aneurysm instead of a valve perforation. The “cleft” edge and the aneurysm were closed.

**Table:** 

Abbreviations, Acronyms & Symbols	
MVA	= Mitral valve aneurysm
TTE	= Transthoracic echocardiography

## INTRODUCTION

Left atrioventricular valve aneurysm is a rare condition^[1]^, being most common in patients with infective endocarditis^[2]^. This aneurysm is usually reported as large and well-defined by transthoracic echocardiography (TTE)^[2]^. Here we present a rare case of partial atrioventricular septal defect with an extremely thin left atrioventricular valve aneurysm mimicking valve perforation. The aneurysm and the “cleft” were closed by an autologous pericardial patch.

## CASE PRESENTATION

A 48-year-old female was diagnosed with partial atrioventricular septal defect in childhood, but she was not followed up. The patient had no previous history of endocarditis or inflammation. She complained about dyspnea associated with chest pain in exercise, but she had no signs of heart failure, and the rest of her examination was normal. Chest X-ray showed cardiomegaly. And three-dimensional transesophageal echocardiography found:

- Ostium primum defect (15 × 35 mm).
- Atrial septal defects with ostium secundum defect (8 mm) with exclusive left to right shunt.
- Right and left atrial dilatation.
- Right ventricular dilatation with right ventricular systolic pressure at 35 mmHg.
- Left atrioventricular valve (Figure 1A) with a significant leak - a predominant central component on the “cleft”.
  **Fig. 1:**
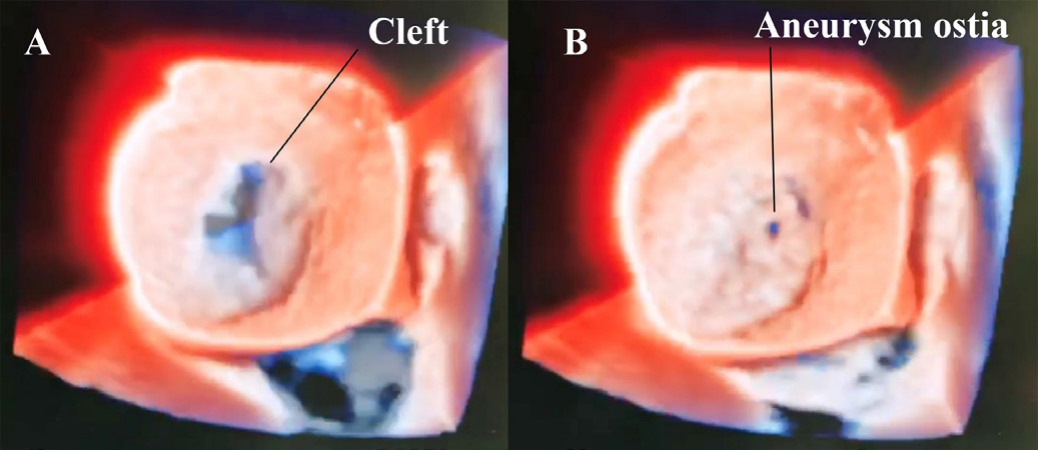
Transesophageal three-dimensional echocardiogram revealing a “cleft” (A) and left atrioventricular valve perforation (B) mimicking perforation by endocarditis.
- On left atrioventricular valve, perforation of 3 mm (Figure 1B) in the inferior bridging leaflet (Video 1).
  **Video 1:**
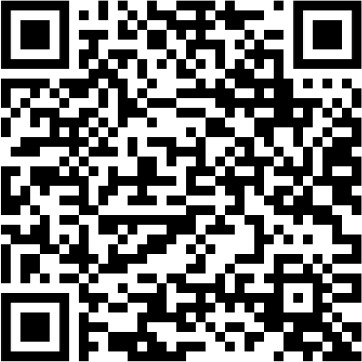
Transesophageal three-dimensional echocardiography clip revealing a “cleft” and left atrioventricular valve perforation mimicking perforation by endocarditis.
- Left atrioventricular valve ring (35 × 39 mm).
- Satisfactory subvalvular apparatus with two papillary muscles.

**Fig. 2:**
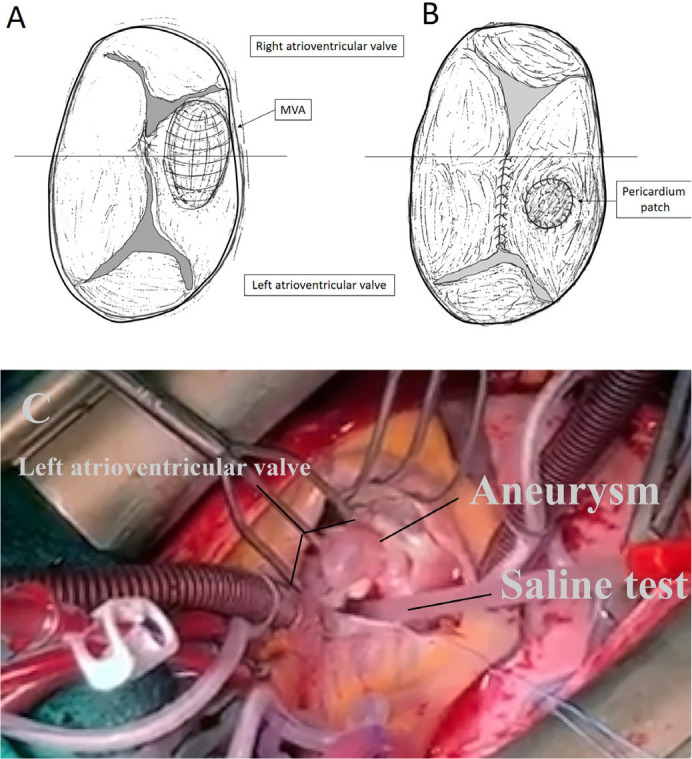
Schema of atrioventricular valve with aneurysm before repair (A) and after repair (“cleft” closure and closed aneurysm with autologous pericardium patch) (B). (C) Intraoperative photograph of the left atrioventricular leaflets with aneurysm. The arrow indicates valve aneurysm. MVA=mitral valve aneurysm.

Coronarography was normal. We performed a median sternotomy with cardiopulmonary bypass and aortic cross-clamping. During direct inspection, we discovered an aneurysm on the left atrioventricular valve, precisely on the inferior bridging leaflet (Figures 2C and 2A). The aneurysm was 5 mm × 7 mm in diameter, and the subvalvular apparatus was normal. Also, the aneurysm was extremely thin (< 1 mm) (Video 2).

**Video 2:**
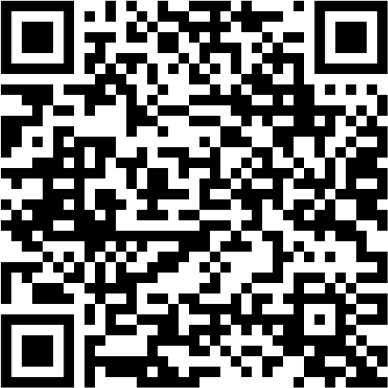
Intraoperative video of the left atrioventricular leaflets with aneurysm. The arrow indicates valve aneurysm.

Edge-to-edge closure of the “cleft” was performed (Figure 2B). The aneurysm had a narrow neck arising from the 3-mm ostium, and it was possible to gather all of the emptied aneurysm and simply cover it with an autologous pericardium patch sutured over the aneurysm with running suture (Figure 2B). The final saline test showed no leakage. Ostium primum and ostium secundum were closed with an autologous patch with running suture. TTE demonstrated no left atrioventricular valve leakage with no aneurysm. The patient’s postoperative course was uneventful, with a normal sinus rhythm. TTE showed no left atrioventricular valve regurgitation and no valve aneurysm at discharge and one year of follow up.

## DISCUSSION

In the literature, there is only one case report who associated a partial atrioventricular septal defect with left atrioventricular valve aneurysm. Imamura et al.^[3]^ reported this association, but the valve aneurysm was a tissue aneurysm closing a ventricular septal defect underneath the atrioventricular valve. Our patient did not have a ventricular septal defect. So, the etiology of this case is probably different from that previously reported. In this clinical case, the assumption was that the left atrioventricular valve regurgitation jet was mainly from this “cleft” that was wide open. We cannot rule out undiagnosed infective endocarditis, but this seems unlikely.

Our lesion anatomically resembled a mitral valve aneurysm, which is a saccular and bulging structure of the mitral leaflet that expands on systole and collapses during diastole. Still, an aneurysm of the mitral valve is rare^[1]^.

Mitral valve aneurysms are usually reported as a sequelae of infective endocarditis^[4,5]^. However, the underlying mechanism for their development is not known. Probably, they are the result of valvulitis with consequent formation of granulation tissue and scar tissue that succumbed to intraventricular pressure with the formation of sac-like outpouchings^[6]^. Mitral valve aneurysm may be induced by connective tissue diseases like Marfan syndrome, osteogenesis imperfecta, and pseudoxanthoma elasticum^[7]^.

Left atrioventricular valve aneurysm is a difficult diagnosis to make by echocardiography and it often mimics a valve perforation due to infectious endocarditis. This mistake could be explained by the particular thinness of the aneurysmal membrane and also a lack of resolution of echocardiography.

We recommend surgical management of a left atrioventricular valve aneurysm because it might be complicated by rupture, thromboembolism, or endocarditis. When left atrioventricular valve repair is not possible due to severely distorted leaflets or too small healthy part of the valve, we suggest left atrioventricular valve replacement^[6]^. The surgical management consists of closing the left atrioventricular valve aneurysm; if the ostium of the valve aneurysm is small (< 3 mm), by direct suture^[8]^; if it is large (> 3 mm), by interposition of an autologous pericardial patch. In our case, the aneurysm was really very thin. We preferred not to resect the excess aneurysm tissue and close the defect with a patch, but to cover the aneurysm with a patch of pericardium, which was sutured to the healthy walls of the valve.

## CONCLUSION

In conclusion, left atrioventricular valve aneurysm with partial atrioventricular septal defect is an unusual case, especially with the aneurysm mimicking valve regurgitation by endocarditis. The autologous pericardium patch can be used to close the large left atrioventricular valve aneurysm.

**Table:** 

Authors’ Roles & Responsibilities	
ML	Substantial contributions to the conception or design of the work; or the acquisition, analysis, or interpretation of data for the work; drafting the work or revising it critically for important intellectual content; final approval of the version to be published
HR	Substantial contributions to the acquisition and analysis of data for the work; final approval of the version to be published
ACC	Substantial contributions to the acquisition and analysis of data for the work; final approval of the version to be published
LM	Drafting the work or revising it critically for important intellectual content; final approval of the version to be published
PA	Drafting the work or revising it critically for important intellectual content; final approval of the version to be published
AT	Drafting the work or revising it critically for important intellectual content; final approval of the version to be published
